# Overlapping Phenotypes in Autism Spectrum Disorder and Developmental Coordination Disorder: A Cross-Syndrome Comparison of Motor and Social Skills

**DOI:** 10.1007/s10803-016-2794-5

**Published:** 2016-04-28

**Authors:** Emma Sumner, Hayley C. Leonard, Elisabeth L. Hill

**Affiliations:** Department of Psychology, Goldsmiths, University of London, New Cross, London, SE14 6NW UK; Department of Psychology, University of Surrey, Guildford, UK

**Keywords:** Autism spectrum disorders, Developmental coordination disorder, Face processing, Motor ability, Social behaviour

## Abstract

Motor and social difficulties are often found in children with an autism spectrum disorder (ASD) and with developmental coordination disorder (DCD), to varying degrees. This study investigated the extent of overlap of these problems in children aged 7–10 years who had a diagnosis of either ASD or DCD, compared to typically-developing controls. Children completed motor and face processing assessments. Parents completed questionnaires concerning their child’s early motor and current motor and social skills. There was considerable overlap between the ASD and DCD groups on the motor and social assessments, with both groups more impaired than controls. Furthermore, motor skill predicted social functioning for both groups. Future research should consider the relationships between core symptoms and their consequences in other domains.

## Introduction

Developing motor skills provides infants with increasing opportunities to interact with the world and the people around them and is, therefore, important in both cognitive and social development (Leonard and Hill 2014). Evidence for relationships between motor and social skills has been reported in typically-developing infants. In particular, fine motor milestones, such as reaching, grasping and manipulating objects, are related to social attention (Libertus and Needham 2010), while changes in posture (i.e., from lying to sitting upright) and in the ability to move around the environment (i.e., crawling and walking) are related to social referencing and interaction (Campos et al. 2000; Clearfield et al. 2008; Clearfield 2011; Karasik et al. 2011). Developing motor skills also provide increasing opportunities for infants to learn about different aspects of faces. For example, infants who begin to move around the room by crawling and walking may be exposed to a range of different facial expressions, including anger or fear, from their parents, which infants who cannot explore the environment are less likely to encounter (Campos et al. 2000). The ability to interpret and act on these facial cues is considered to be central to social competence (Lemerise and Arsenio 2000), and therefore infants who have delays in early motor milestones could be at risk for problems in a range of social outcomes.

Two neurodevelopmental disorders in which motor difficulties have been highlighted are autism spectrum disorder (ASD) and developmental coordination disorder (DCD). ASD is diagnosed on the basis of difficulties in social functioning, along with restricted patterns of behaviour and interests; while a diagnosis of DCD results from motor coordination difficulties which have a significant impact on activities of daily living and academic achievement (APA 2013). Although classified as discrete disorders under the current diagnostic framework (American Psychiatric Association [APA] 2013), research has suggested that the two disorders share some characteristics. An increasing number of studies recognize motor difficulties in individuals with ASD, including infants at increased genetic risk of developing the disorder (e.g., Lloyd et al. 2013; Leonard et al. 2014a; Bhat et al. 2011 for a review). Furthermore, peer difficulties and social problems have been identified in individuals with DCD (Chen et al. 2009; Cummins et al. 2005; Dewey et al. 2002; Wagner et al. 2012). Children with DCD have been reported to spend more time on their own and less in large group activities, especially physical ones (Poulsen et al. 2007; Smyth and Anderson 2000), and have higher levels of parent-reported social problems (Chen et al. 2009; Cummins et al. 2005; Dewey et al. 2002).

However, relatively few studies have specifically investigated the relationship between motor and social skills in the two named disorders. Those that have are usually focused on a different timeframe from studies of typical development because ASD and DCD are not diagnosed reliably before the age of 2 or 5 years, respectively (Charman and Baird 2002; Blank et al. 2011). Studies of school-aged children with ASD have reported significant correlations between motor skill and degree of parent-reported social impairment (Dyck et al. 2007; Hilton et al. 2011; Hirata et al. 2014; MacDonald et al. 2013); and in a recent study of pre-school children, fine motor functioning was related to language and social orientation during object exploration (Hellendoorn et al. 2015).

Difficulties in encoding and using information provided by faces (such as identity, gaze and emotional expressions) have long been reported in ASD (e.g., Annaz et al. 2009; Ashwin et al. 2015; Dawson et al. 2005; Harms et al. 2010; Riby et al. 2008; Wallace et al. 2008), and these difficulties may explain some of the social problems seen in the disorder (Adolphs et al. 2001; Corbett et al. 2014). One study investigating infants at-risk of ASD has considered the relationship between face processing ability and early motor skills Leonard et al. (2014b). In this study, at-risk infants who had poor motor skills at 9 months, as assessed by parent report, performed more poorly on face processing tasks at 5–6 years, even though they had not been diagnosed with ASD in the intervening period. Only one study, to our knowledge, has assessed face processing in DCD. Cummins et al. (2005) reported significantly poorer recognition of facial emotions in children with DCD when compared to age-matched controls, although the relationship between motor and face processing difficulties was not directly assessed. As in ASD, the authors suggested that difficulty processing social cues from the face may provide a pathway to the peer problems reported in children with DCD.

Notably, little research has been conducted to directly compare the difficulties presented in the two disorders. The current study aimed to address this gap in the literature by directly comparing the motor and social abilities of children with ASD, children with DCD, and a typically-developing (TD) age-matched control group and, in turn, exploring the relationship between motor and social functioning in more detail than has been done previously. Parent-report questionnaires relating to early motor and current motor abilities and social skills were used alongside performance-based measures of motor and social skills. The analyses aimed to address the following research questions: (1) Do parents report delayed achievement of motor milestones in children with ASD and DCD, compared to parents of TD children? (2a) Are school-aged children with ASD and DCD impaired in both motor and social skills in comparison to TD children? and (2b) Can the three groups be distinguished from each other based on these abilities? (3) Does the relationship between motor and social skills differ between all three groups?

It was expected that a greater proportion of those with DCD and ASD would be delayed in achieving motor milestones than the TD group, and that those children with a neurodevelopmental disorder would have lower motor functioning scores than TD children. While children included in the ASD group did not have a diagnosis of DCD, based on existing findings in the literature reporting substantial movement impairments in a sample of adolescents with ASD (Green et al. 2009), it was expected that the ASD group motor scores would fall between the TD and DCD groups. Given that problems with processing facial expressions have been reported previously in children with ASD and DCD, it was predicted that both groups would perform more poorly on facial expression processing compared to TD controls, while difficulties in processing identity and eye gaze were also expected in the ASD group. Furthermore, it was hypothesized that children in the ASD and DCD groups would demonstrate weaknesses in social functioning compared to their TD counterparts, with the ASD group performing more poorly overall.

Finally, the relationship between the motor and social tasks was compared between groups. Specifically, we aimed to investigate whether and to what extent motor skills were related to social functioning in every group, and whether there were differences in this relationship between groups. Based on the research of children with ASD and infants at-risk of ASD reviewed above, it was predicted that motor abilities would be a significant predictor of the variance in parent-reported social skills and face processing performance. It was unclear whether the relationship between motor and social skills would be evident in the TD group within this age range, or in the DCD group.

## Methods

### Participants

Thirty children with ASD (25 boys) and 30 children with DCD (21 boys) were compared to 35 TD children (26 boys): all groups were aged 7–10 years. Group characteristics are presented in Table 1. A one way analysis of variance (ANOVA) revealed no significant group differences in age of participants, *F*(2,92) = 2.22, *p* = .12; further confirmed by post hoc comparisons. Demographic information was gathered for all participants. Parental education has been used as a measure of socio-economic status in similar studies (Fernald et al. 2013; LeBarton and Iverson 2013). Parental education was found to be comparable across all three groups.

**Table 1:** Background characteristics of the three groups

	TD (*n* = 35)	ASD (*n* = 30)	DCD (*n* = 30)
Gender (m; f)	26; 9	25; 5	21; 9
Age in years			
Mean (SD)	9.11 (.95)	8.65 (1.18)	8.61 (1.16)
Range	7.50–10.74	7.01–10.91	7.04–10.90
Maternal education			
Mean (SD)	4.85 (1.45)	4.70 (1.46)	5.13 (1.18)
Range	2–7	1–7	2–7
Paternal education			
Mean (SD)	5.18 (1.31)^a^	4.14 (1.53)^a^	4.86 (1.55)
Range	2–7	1–7	2–7

Parental education was scored based on the education system on a scale from 1 (no qualifications) to 7 (qualified to doctoral level). Scores of 4 and 5 represent further education and degree level status, respectively

^a^ *n* = 2 missing data points

Set inclusion criteria that applied across all groups required that Full Scale IQ (FSIQ) was above 70. In addition, prior to recruitment, all children in the clinical groups had an existing diagnosis (of either ASD or DCD) from relevant clinicians independent of the research study.

Children with ASD were recruited through an advertisement placed with a charitable foundation, the National Autisitc Society (UK), as well as through local schools in South London with specialist units or provision for students with ASD. An ASD diagnosis was corroborated by a member of the research team trained to administer the Autism Diagnostic Observation Schedule (ADOS-2; Lord et al. 2012). This is a semi-structured observation using tasks that tap certain behaviours, such as conversation and reciprocal social interaction. Module 3 of the ADOS-2 was appropriate for all participating children with ASD. Of note, 5 of the children in the ASD group were unable to complete the ADOS in the study protocol because they had recently undergone this assessment as part of their formal diagnosis. However, the parents of these children provided the clinician’s report detailing their child’s performance on the ADOS and completed the Social Communication Questionnaire (SCQ; Rutter et al. 2003) to confirm ASD-related symptomatology (see “Materials” for task details). All remaining children scored above cut-off on the ADOS-2, demonstrating a total mean score of 8.52 (standard deviation 1.09). Parents also completed a background screening questionnaire, confirming that children in this group did *not* have a co-occurring diagnosis of DCD.

Children with DCD were recruited through an advertisement placed with a charitable foundation, the Dyspraxia Foundation (UK). All children met the Diagnostic and Statistical Manual (DSM-5) criteria for DCD (APA 2013). The research team confirmed that children with DCD had significant motor difficulties, scoring at or below the 16th percentile on the MABC-2 (Henderson et al. 2007). On the screening questionnaire, parents confirmed that there was no history of additional diagnoses, such as Attention-Deficit-Hyperactivity-Disorder (ADHD), language impairment or ASD, neurological impairment, or a medical condition which might explain the child’s motor impairment.

The TD group was recruited through local primary schools in South London. Parents of the children in this group did not identify diagnoses of any neurodevelopmental disorders. Moreover, to eliminate motor and social difficulties, all children scored at or above the 25th percentile on the motor assessment (MABC-2), and below cut-off for ASD on the SCQ, respectively.

### Materials

#### Inclusion Measures

*Intellectual ability* was measured using the Wechsler Intelligence Scale for Children (WISC-IV, UK norms; Wechsler 2003). FSIQ (*M* = 100, *SD* = 15) is the sum of the four indices: verbal comprehension, perceptual reasoning, working memory and processing speed. Ten subtests are split across the four indices, all of which were completed by each child.

*Motor competency* was measured using the Movement Assessment Battery for Children, second edition, age band 2 (7–10 years) (MABC-2; Henderson et al. 2007), which is a standardised assessment comprising three components: manual dexterity (3 items), aiming and catching (2 items), and static and dynamic balance (3 items). Summing all scores yielded a total standard score (*M* = 10, *SD* = 3) and percentile rank (UK norms). Percentile ranks are used to identify those with ‘significant’ (5th percentile) or ‘borderline’ (16th percentile) motor coordination difficulties. As all children in the DCD group had an existing diagnosis, those that scored on the 16th percentile (*n* = 2) were included in the sample. As part of the inclusion criteria, all children in the TD group had to score at or above the 25th percentile. No cut-off was specified for the ASD group; therefore, in this respect, the MABC-2 served as an exploratory measure of motor skill.

The SCQ is a parent-report screening measure of *ASD*-*related symptomology*. The ‘lifetime’ version was used, which consists of 40 questions about current behaviour and behaviour during the period of time between the child’s 4th and 5th birthday. Scores above a cut-off score of 15 are suggestive of ASD. The SCQ was used to further confirm the diagnosis of the ASD group, and to ensure that the TD group did not present with ASD-related social communication difficulties.

#### Performance Measures

##### Motor Ability

*Early motor abilities* were assessed by parents’ responses to a motor milestones questionnaire (adapted from Brouwer et al. 2006), which asked the age (in months) at which their child first achieved various key milestones. Parent-report was retrospective and, therefore, only the milestones that were most confidently reported are included in the study; these were when their child first crawled on hands and knees, stood unassisted, and walked unassisted.

Parents completed the Vineland Adaptive Behavior Scales questionnaire (VABS-II; Sparrow et al. 2005), which measures current abilities, requiring parents to report whether their child ‘Never’, ‘Sometimes’ or ‘Usually’ demonstrates a particular behaviour. The *Gross and Fine Motor* scales were used to assess motor functioning. Standard scores were not available for these scales for the present age range; therefore, raw scores are provided.

##### Social Functioning

The Benton Test of Facial Recognition (hereafter, “Benton”; Benton et al. 1983) uses a face matching paradigm to assess *identity recognition.* The short form was used, which has 13 items and a maximum score of 27. For items 1–6, children must identify the target photograph (face) out of six alternative photographs shown on a separate page (all frontal view). For items 7–13, the child must identify three out of the six alternatives that are the ‘same person’ as the target: this time the photographed individuals are facing a different angle, or with different lighting. The total number of correct answers was calculated.

The battery of *face processing* tasks developed by Bruce et al. (2000) was developed for children aged 4–10 years. Greyscale images of children’s faces were presented on a laptop. For the purpose of this study, the ‘match’ tasks of the battery were used, as they were found by the authors to be most appropriate for the identified age groups. Children had to identify which face (out of 2) in the bottom row (a) felt the same (expression identification), (b) was making the same sound (speech sound/lip reading), or (c) was looking in the same direction (eye gaze detection), as the person pictured on the top row. Each test comprised of 12 items. Scores were converted to percentages, representing accuracy.

The socialization domain from the VABS-II (Sparrow et al. 2005) was included as a measure of *social functioning*. Socialization scores are calculated from questions in the following components: play and leisure time, interpersonal relationships and coping skills. Raw scores were transformed to v-scale scores for each component, and combined to produce an overall standard score (*M* = 100, *SD* = 15).

### Procedure

The present study was approved by the Goldsmiths, University of London, ethics committee. Written informed consent was obtained from all parents/carers. Children in the ASD group completed the tasks over three sessions, which took place at either the research lab, during a home visit, or at their school. The DCD group completed the tasks over one or two sessions, which took place at the lab or their home. The TD group was tested at their school across two sessions. Children were seen individually in a quiet room. In all cases, the motor assessment was completed first, followed by the WISC and the social measures. Parents chose to either complete the questionnaires during the testing session or to send the completed packs to the research team. Assessments were administered according to the procedures identified in the test manuals.

### Data Analysis

In a few cases, parents did not return or fully complete the questionnaires. Namely, 2 parents from each group did not complete the motor milestones, 1 parent from each group did not complete the SCQ or the VABS; while an additional parent from the TD group completed only part of the VABS (socialisation, but not the motor questions). All missing data points are marked in the corresponding tables. If data were normally distributed, ANOVAs and post hoc comparisons were used to compare groups. When first exploring the inclusion measures, the non-parametric Kruskal–Wallis test was conducted on data that violated parametric assumptions. After identifying possible variables to control for from these initial analyses, data that were not normally distributed were analysed using the robust method of bootstrapping (Field 2013) in later analyses (Tables 3, 4). This enabled comparisons across all three groups and the inclusion of necessary covariates, which is not possible in non-parametric tests. A series of regression analyses were conducted to compare the relationship between motor and social abilities across groups, also using the bootstrapping method.

## Results

The results are shown in four sections. First, the inclusion criterion is addressed, and then the remaining sections explore the three research questions. Table 2 reports the inclusion measures. All children demonstrated a FSIQ above cut-off (70). An ANOVA revealed significant group differences in FSIQ, *F*(2,91) = 10.14, *p* < .001, η_*p*_^2^ = .18, with Gabriel post hoc tests confirming TD children scored significantly higher than the ASD and DCD groups; while the two clinical groups were comparable (*p* = .46).

**Table 2:** Mean (SD) and range of scores for the inclusion measures for the three groups

	TD (*n* = 35)	ASD (*n* = 30)	DCD (*n* = 30)
FSIQ standard score			
Mean (SD)	108.22 (10.13)	101.03 (14.62)	98.43 (13.14)
Range	89–127	82–127	81–126
MABC-2 percentile			
Mean (SD)	64.80 (22.07)	30.22 (31.92)	3.48 (4.82)
Range	25–98	.01–95	.01–95
SCQ raw score			
Mean (SD)	2.79 (2.58)^a^	22.52 (6.08)^a^	9.79 (6.19)^a^
Range	0–7	15–38	1–27

FSIQ = Full Scale IQ; Standard Score *M* 100, *SD* 15; MABC-2 = Movement Assessment Battery for Children; SCQ = Social Communication Questionnaire

^a^1 missing data point

The MABC-2 confirmed that all TD children scored at or above the 25th percentile. Of note, 2 (7 %) children with DCD scored on the 16th percentile, while the remaining DCD participants scored on the 9th (*n* = 6, 20 %) or below the fifth (*n* = 22, 73 %). Although not an inclusion measure for the ASD group, 16 children with ASD (53 %) scored at or below the 16th percentile. The Kruskal–Wallis test revealed that motor skill was significantly different across groups, *H*(2) = 56.62, *p* < .001. Mann–Whitney tests confirmed the DCD and ASD groups scored significantly lower than the TD group, *U* = 465.00, Z = −6.93, *p* < .001, *r* = −.89; *U* = 207.50, Z = −4.20, *p* < .001, *r* = −.52, respectively; while children with DCD had poorer motor skill than the ASD group, *U* = 154.00, Z = −4.41, *p* < .001. *r* = −.56.

The SCQ results confirmed that all children with ASD scored above the cut-off of 15 and all TD children scored below cut-off. Only 5 children with DCD (17 %) scored above the SCQ cut-off. Significant group differences were found for the SCQ, *F*(2,89) = 136.70, *p* < .001, η_*p*_^2^ = .76. Post hoc analyses (all *p* < .001) revealed that the TD group had significantly fewer autism-related symptoms than the DCD and ASD groups, with the DCD group also showing significantly fewer symptoms than the ASD group.

1. Do parents report delayed achievement of motor milestones in children with ASD and DCD, compared to parents of TD children?

**Fig. 1:**
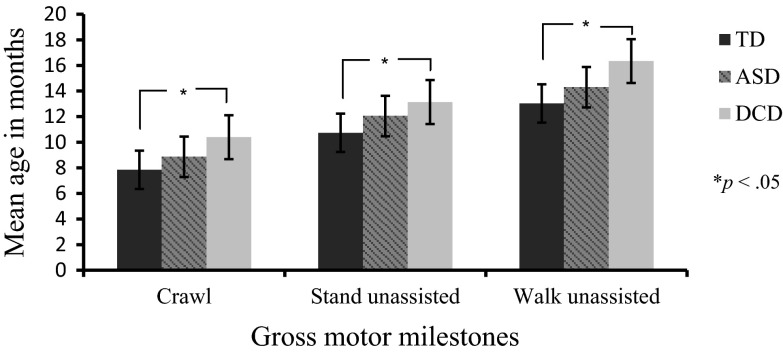
Mean age (+S/E bars) of motor milestone achievements

Figure 1 displays the average age at which parents reported their child to have first crawled, stood unassisted, and walked alone. Although 33 parents in the TD group, 28 in the ASD and 28 in the DCD group returned the questionnaire, some parents left the boxes blank when they failed to recall when the milestone occurred. Percentage of parental recall success was: TD: 85 % for crawling, 90 % for standing, 94 % for walking; ASD: 75 % crawling, 71 % standing, 79 % walking; DCD: 86 % crawling, 86 % standing, 93 % walking. Seven parents of children with DCD (23 %) reported that their child did not crawl at all, as did 2 parents of children with ASD (7 %); in contrast to the TD children who all acquired this skill.

A mixed ANOVA revealed there was a significant main effect of the time at which milestones were achieved, *F*(2, 120) = 201.80, *p* < .001, η_*p*_^2^ = 77, and a significant effect of group, *F*(2, 62) = 6.36, *p* = .003, η_*p*_^2^ = .17. Pairwise comparisons revealed no significant differences between the TD and ASD groups (*p* = .09) or the ASD and DCD groups (*p* = .61); however, differences were found between the TD and DCD groups (*p* < .001). Lack of an interaction, *F*(4, 124) = .44, *p* = .78, η_*p*_^2^ = .01, indicated all groups completed the milestones in the same order.

- Are school-aged children with ASD and DCD impaired in both motor and social skills in comparison to TD children?
- Can they be distinguished from each other based on these abilities?

Performance on the motor and social measures for each group is shown in Table 3. Measures were bootstrapped and analysed using univariate analyses of covariance (ANCOVAs), including FSIQ as the covariate unless otherwise noted. Age was not included as a covariate in these analyses of group differences, as the three groups were matched by mean age and no significant correlations were found between age and the reported measures in Table 3. Significance values were based on Bonferroni-corrected values for multiple comparisons, *p* = .006. After controlling for FSIQ, there was a significant effect of group on motor competency as measured by the MABC-2 total standard score, *F*(2, 91) = 52.74, *p* < .001, η_*p*_^2^ = .54, VABS Gross, *F*(2, 87) = 25.13, *p* < .001, η_*p*_^2^ = .37, and VABS Fine motor skill, *F*(2,87) = 24.89, *p* < .001, η_*p*_^2^ = .36. Post-hoc tests revealed the DCD and ASD groups scored lower than the TD group on all motor measures (*p*s < .001). The ASD and DCD groups were similar on the VABS Gross motor scores (*p* = .15) and VABS Fine motor scores (*p* = .15) but, as before, the DCD group scored lower than the ASD group on the MABC-2 (*p* < .001).

**Table 3:** Mean (SD) and range of scores for the three groups on all motor and social measures

Measures	TD (n = 35)	ASD (n = 30)	DCD (n = 30)	Post hoc
*Motor ability*				
MABC-2 Total SS				
Mean (SD)	11.40 (2.07)	7.50 (3.74)	3.37 (1.99)	TD > ASD > DCD
Range	8–16	1–15	1–7	
VABS Gross Motor Raw Score^†^				
Mean (SD)	79.60 (1.14)^b^	72.55 (6.71)^a^	69.89 (4.75)^a^	(ASD = DCD) < TD
Range	75–80	60–80	60–78	
VABS Fine Motor Raw Score^†^				
Mean (SD)	70.73 (1.86)^b^	60.65 (9.70)^a^	57.21 (6.33)^a^	(ASD = DCD) < TD
Range	65–72	46–75	43–71	
*Social functioning*				
Benton Raw Score				
Mean (SD)	21.77 (2.39)	19.73 (2.64)	19.03 (2.44)	(ASD = DCD) < TD
Range	14–26	14–24	12–24	
Bruce Expression Match				
Mean % correct (SD)	96.67 (6.13)	92.22 (10.48)	87.22 (14.48)	(ASD = DCD) < TD
Range	75–100	59–100	42–100	
Bruce Speech Sound Match				
Mean % correct (SD)	97.86 (4.21)	91.38 (9.15)	89.72 (9.45)	(ASD = DCD) < TD
Range	83–100	75–100	67–100	
Bruce Gaze Match				
Mean % correct (SD)	97.71 (6.45)	88.66 (17.37)	91.00 (13.98)	(ASD = DCD) < TD
Range	70–100	40–100	50–100	
SCQ Total Score^†^				
Mean	2.79 (2.58)	22.51 (6.08)	9.79 (6.19)	TD < DCD < ASD
Range	0–7	15–38	1–27	
VABS Socialisation SS^†^				
Mean (SD)	107.32 (14.12)^a^	73.28 (12.86)^a^	87.28 (12.35)^a^	TD > DCD > ASD
Range	83–135	50–117	68–117	

Post hoc results are after controlling for FSIQ, apart from for the Bruce measures

SS = Standard Score; MABC-2 = Movement Assessment Battery for Children, Standard scores = *M* 10, *SD* 15, VABS = Vineland Adaptive Behaviour Scales, Standard scores = *M* 100, *SD* 15. SCQ = Social Communication Questionnaire

^a^1 missing data point

^b^2 missing data points

^†^Parent report

Analyses of the Benton measure of face processing revealed a significant effect of group after controlling for FSIQ, *F*(2,91) = 5.28, *p* = .01, η_*p*_^2^ = −.10. Post-hoc tests demonstrated that children with DCD scored significantly below the TD group (*p* = .002), as did the ASD group when compared to the TD group (*p* = .01). Comparisons of the ASD and DCD groups revealed no significant differences (*p* = .44) on the Benton. The Bruce measures of face processing violated assumptions for parametric tests even after bootstrapping data.^1^ Kruskal–Wallis tests revealed significant group differences for Expression Match, *H*(2) = 11.73, *p* = .003, and Sound Match, *H*(2) = 19.26, *p* < .001, but not Gaze Match, *H*(2) = 8.92, *p* = .02, when employing the strict Bonferroni correction. Mann–Whitney tests followed up these group comparisons. The ASD and DCD groups scored significantly lower than the TD group on the Expression Match task, *U* = 395.00, Z = −1.96, *p* = .03, *r* = −.42 (ASD), *U* = 290.50, Z = −3.38, *p* < .001, *r* = −.42 (DCD); the Sound Match task, *U* = 304.50, Z = −3.32, *p* < .001, *r* = −.41 (ASD), *U* = 233.00, Z = −4.26, *p* < .001, *r* = −.53 (DCD); and the Gaze Match task, *U* = 361.50, Z = −2.73, *p* = .003, *r* = −.34 (ASD), *U* = 368.00, Z = −2.63, *p* = .004, *r* = −.33 (DCD). Comparison of the ASD and DCD groups revealed no significant differences on any of these tasks, *U* = 354.00, Z = −1.49, *p* = .14, *r* = −.44 (Expression Match), *U* = 398.50, Z = −.80, *p* = .42, *r* = −.10 (Sound Match), and *U* = 431.50, Z = −.30, *p* = .76, *r* = −.04 (Gaze Match).

The final parent-report social measures in Table 3 met ANCOVA assumptions. After controlling for FSIQ, there was a significant effect of group on autism-related symptomology as measured by the SCQ, *F*(2, 88) = 108.21, *p* < .001, η_*p*_^2^ = .71, and social functioning as measured by the VABS Socialisation, *F*(2, 88) = 43.74, *p* < .001, η_*p*_^2^ = .50. Post hoc tests revealed a similar pattern for SCQ and VABS measures, in that both the ASD and DCD groups were significantly more impaired (scoring higher on the SCQ, lower on the VABS; corresponding with rating scales) than the TD group (*p* < .001). Direct comparison of the ASD and DCD groups demonstrated significant group differences, with the ASD group being identified by parents as presenting with more difficulties than the DCD group (*p* < .001).

3. Does the relationship between motor and social skills differ between groups?

To answer the final research question, motor ability was entered as a predictor of three social outcome measures, namely the Bruce Expression Match, Bruce Gaze Match, and VABS Socialisation scale (one regression for each task). Due to the VABS Gross and Fine motor scores being highly correlated, and thus violating the assumption of multicollinearity for regression analyses, the two scores (Gross and Fine) were combined to form a ‘VABS Motor Composite’. This composite score was used as the motor predictor variable for each regression.^2^ Further, age was entered as a predictor and also FSIQ because the groups differed slightly on the latter measure.

Only three outcome measures were selected to reduce the number of statistical comparisons made. These variables included measures of face processing (Bruce; expression and gaze) and social functioning in the broader sense (VABS Socialization domain). The VABS manual highlights that the gross and fine motor scales are moderately correlated with the Socialization domain (*r* = .44 and .56, respectively) for children aged 2–6 years of age. These data were unfortunately not available for the age range used in the present study; however these correlations should be kept in mind when interpreting the regression results. The Bruce measures were selected on the basis of the results in Table 3 whereby, although not statistically significant, the mean scores demonstrated relatively weaker performance on Expression Match for those with DCD compared to the ASD group, and on Gaze Match for those with ASD compared to the DCD group. A group variable was entered into Step 2 of each regression in order to compare groups on the relationships between motor and social skills. Thus, for each of the three outcome measures, three regressions were conducted in order to make all possible group comparisons (TD vs. ASD, TD vs. DCD, ASD vs. DCD), resulting in a total of 9 regressions (the significance of each final model was assessed against a Bonferroni-corrected value of *p* = .006). Summary details of these regressions are provided in Table 4. To remain concise, only significant results are discussed.

**Table 4:** Summary of regression analyses predicting performance on three key social measures

Social measure	Final model Adjusted R^2^	Step 2 for each regression				
		Age	*β*	*Β*	*β*	*∆R* ^*2*^
			FSIQ	VABS Motor Composite	Group	
Bruce Expression						
TD versus ASD	.08	.05	.16	.05	−.15	.01
	*p* = .28	*p* = .77	*p* = .19	*p* = .78	*p* = .41	*p* = .40
TD versus DCD	.22	.19	.19	−.02	.28	.02
	*p* = .01	*p* = .11	*p* = .13	*p* = .91	*p* = .19	*p* = .25
ASD versus DCD	.09	.16	.19	−.01	.18	.03
	*p* = .24	*p* = .24	*p* = .07	*p* = .92	*p* = .15	*p* = .19
Bruce Gaze Match						
TD versus ASD	.23*	−.17	.10	.38	−.11	.01
	*p* = .005	*p* = .36	*p* = .49	*p* = .18	*p* = .53	*p* = .51
TD versus DCD	.19	.28	.22	−.14	.26	.02
	*p* = .02	*p* = .05	*p* = .22	*p* = .67	*p* = .42	*p* = .30
ASD versus DCD	.07	.01	.13	.19	−.15	.02
	*p* = .45	*p* = .97	*p* = .39	*p* = .31	*p* = .35	*p* = .29
VABS Socialisation						
TD versus ASD	.67**	−.12	−.02	.27*	−.66**	.24**
	*p* < .001	*p* = .22	*p* = .78	*p* = .01	*p* < .001	*p* < .001
TD versus DCD	.47**	−11	.15	.49**	.14	.01
	*p* < .001	*p* = .31	*p* = .19	*p* < .001	*p* = .36	*p* = .48
ASD versus DCD	.47**	−.13	.09	.42*	−.60**	.33**
	*p* < .001	*p* = .06	*p* = .36	*p* = .01	*p* < .001	*p* < .001

For each regression, Age, FSIQ and VABS motor composite scores were entered at Step 1 (of note, this step of each model is not shown for brevity), then Group (TD vs. ASD, TD vs. DCD, ASD vs. DCD) was entered in a block at Step 2. The total adjusted *R* ^*2*^ accounted for by the final model is shown in the table. Standardised coefficients are provided for each predictor in Step 2, above significance values from 1000 bootstrapped samples. *∆R* ^*2*^ represents the change in *R* ^*2*^ with the addition of Step 2 (Group). Significance is shown in bold (* *p* < .05, ** *p* < .001). Bonferroni correction of *p* = .006 is applied to the final model. VABS = Vineland Adaptive Behaviour Scale. 5 missing data points for VABS measures (2 TD, 1 ASD, 1 DCD)

After taking into consideration the Bonferroni correction, no significant results were evident for the Bruce Expression Match regressions. Only the Bruce Gaze Match regression comparing the TD and ASD groups was significant, *F*(4, 57) = 4.16, *p* = .005, predicting 23 % of the variance overall, but motor skill and group membership were not significant predictors.

**Fig. 2:**
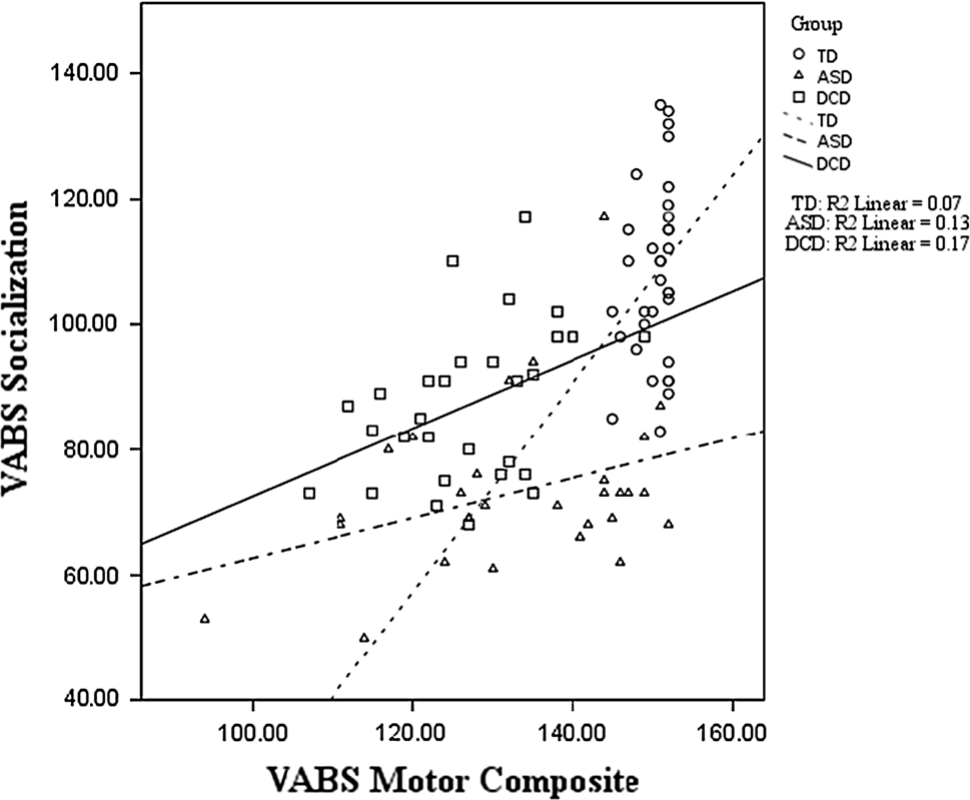
VABS Motor Composite and Socialization scores for each group

All regressions models were significant for the VABS Socialization outcome. Comparing the TD and ASD groups, *F*(4, 57) = 28.33, *p* < .001, the model predicted 67 % of the variance overall. The TD and DCD comparison, *F*(4, 57) = 12.54, *p* < .001, revealed the final model predicted 47 % of the variance; while for the ASD and DCD comparison, *F*(4, 53) = 11.76, *p* < .001, the model predicted 47 % of the variance. As can be seen in Table 4, age and FSIQ were not found to be significant predictors. However, motor ability was a significant predictor in each regression. Including the group comparison at Step 2 resulted in a better model fit overall (represented by a significant change in R^2^ at Step 2, *p* = .05) for only the TD versus ASD and the ASD versus DCD analyses; and the group standardized co-efficient was significant in both cases, indicating that group differences still existed after accounting for the role of motor skill on socialization. Interestingly, group differences did not remain between TD and DCD children after controlling for the role of motor skill on socialization, suggesting that any impact of group was already accounted for by the variable entered at the previous step (i.e., motor ability). Investigating this further, Fig. 2 shows the relationship between motor ability and VABS socialization for each group. For TD children, the correlation between motor and social skills was not significant (*r* = .26, *p* = .14), and only 7 % of the variance in socialization was explained by motor performance.

However, the relationship between motor and social skill was found to be significant for children with ASD (*r* = .36, *p* = .05), and children with DCD (*r* = .41, *p* = .02). Figure 2 shows a linear trend whereby for the ASD group 13 % of the variance in socialization scores was explained by the motor composite; this rose to 17 % for the DCD group.

## Discussion

To the best of our knowledge, the present study provides the first comprehensive account, and direct comparison, of the motor and social abilities of children with ASD, DCD, and TD children. A rigorous method of confirming diagnoses, and eliminating additional co-occurring diagnoses, was employed and the groups were tightly matched for SES and overall mean age.

The first research question addressed early motor skill. The findings highlighted how children with DCD, and to a lesser extent, children with ASD, can be distinguished from their peers in terms of early motor development. Both clinical groups were reported to, on average, reach key motor milestones (crawling, standing, walking) later than their peers, although this delay was only significant for children with DCD. It was noteworthy that 23 % of children in the DCD group and 7 % of those in the ASD group did not learn to crawl at all. Crawling and walking independently, both of which were delayed in comparison to the TD group, enables self-initiated exploration of the environment from a young age (Clearfield 2011). As well as providing opportunities for the child to make decisions based on movement, and strengthening bilateral coordination and muscle tone in the process, early movement has implications for social development (Kretch et al. 2014). Therefore, the next step was to determine whether children with ASD or DCD were distinguishable from their peers in current motor and social performance, and whether there was any notable overlap across the two disorders.

As anticipated, those with a core motor impairment (DCD) performed consistently worse on all motor measures. Children with ASD also performed significantly poorer than their TD peers on the motor assessment and were rated as similar to the DCD group on the VABS Gross and Fine motor questions. Furthermore, over half of the ASD group met the cut-off for motor difficulties on the MABC-2 (Henderson et al. 2007). These observations suggest that co-occurring motor problems are evident in a substantial proportion of the ASD population, and provide support for previous findings of motor difficulties in this group (Green et al. 2009; McPhillips et al. 2014).

Similarly, children with ASD and those with DCD were noticeably worse on the face processing measures compared to their TD peers, who consistently outperformed the two clinical groups in line with our predictions. The fact that the ASD and DCD groups performed similarly to each other on both the Benton face processing measure and all of the Bruce measures (expression, speech sound and gaze; Bruce et al. 2000) suggests that children with DCD do have problems with processing social information. While it was anticipated that the ASD group would have difficulties with face processing (e.g., Adolphs et al. 2001; Harms et al. 2010), it was not predicted initially that the DCD group would perform so similarly on these measures. The parental-report measures further add to the performance-based assessments by providing an indicator of social functioning in the broader sense (e.g., interacting with peers). Children in the ASD group were rated as scoring more poorly than both the TD and DCD groups on the SCQ and VABS Socialization, as predicted, with the DCD group scoring at an intermediate level between the TD and ASD groups. While five children with DCD scored above the cut-off on the SCQ, difficulties reported by parents in socialization in the DCD group were not generally as marked as those seen in the ASD group.

The final stage of analysis aimed to determine the specific relationship between motor and social abilities. While motor skill was not found to predict face processing abilities, in terms of expression or gaze matching, it would appear that motor skill has predictive value (predicting between 27 and 49 % of the variance in all regressions) in relation to socialization (i.e., relating to peers, play and leisure time). Follow up analyses on each individual group revealed that motor skill was significantly correlated with social behaviour for only the ASD and DCD groups, predicting 13 and 17 % of the variance, respectively. Collectively, these findings highlight that while overlapping characteristics are evident in the ASD and DCD groups, motor skill plays a slightly more pronounced role in influencing social behaviour in children with DCD. This motor and social link has been suggested in previous studies of children with DCD (Dewey et al. 2002; Smyth and Anderson 2000) but had not been specifically tested previously. However, it is noteworthy that other factors would appear to be influencing social behaviour to a certain degree in all groups, as a large proportion of variance is left unexplained.

The lack of a relationship between motor and social skills in the TD group should be treated with a degree of caution, as it was noted that many children in this group were performing at ceiling on the motor scales. Thus, a restricted range of motor scores for TD children could partly explain this result. Moreover, a lack of a relationship between motor skill and face processing abilities in general could be due to the age range. It may be that the link is tighter in early development, as infants start to explore their environment and interact with others (Campos et al. 2000). The dynamic systems framework suggests that the motor and cognitive systems follow a similar developmental timetable in early childhood (Diamond 2000). The current findings provide support for this interrelatedness across systems in ASD and DCD populations. In this sense, a practical implication of the findings can be raised in terms of support. There is a clear need to consider a wide range of functioning when working with children with ASD or DCD, and not to focus solely on the diagnostic criteria, to be able to identify possible secondary consequences of the known ‘core’ disorder or even undiagnosed co-occurring difficulties. Taking an all-encompassing approach will ensure the child’s full range of needs can be targeted appropriately and it may be that improvement in one area will have repercussions for another. Related to intervention, the findings suggest that delayed achievement of motor milestones could be used as an early marker of later motor difficulties.

Although the findings provide a comprehensive profile of each group, limitations can be identified. Some parents struggled to retrospectively recall the time at which their child completed a particular motor milestone. In any cases where the parent was unsure, these data were left blank. If a full dataset had been collected this measure could have been used to determine the relationship between early motor skill and later outcomes. Future research could aim to gather these data prospectively or focus on a younger age group, in the hope that parents can recall this early information more easily. Another limitation of the study was the use of scales from the same parent questionnaire—VABS motor and socialization domains—in the regression analyses that explored the relationship between motor and social skills. As highlighted in the results section, these two domains have been shown to be moderately correlated for younger children in validation studies of the VABS. Therefore, future research would benefit from considering other measures of motor skill and their relation to social functioning, and extending this work further to consider these relations over time, rather than at one time-point. Distinctions between the role of fine and gross motor abilities in relation to social skills would also be of interest.

To conclude, using a cross-syndrome comparison approach revealed overlapping profiles in ASD and DCD, despite the two groups being identified as ‘pure’ cases (i.e., no co-occurring diagnoses). However, as a whole, the two disorders remain distinct in the severity of their core difficulty; namely children with ASD are rated as lower in social functioning and children with DCD present with more pronounced motor difficulties. Nevertheless, motor skill has a significant impact on social behaviour for children with DCD and, to a lesser extent, children with ASD. The identification of motor problems in early development could therefore have an important impact on later motor and social skills, and could provide opportunities for earlier intervention for those at risk of developmental difficulties.

## Footnotes

1 Levene’s test revealed unequal variances. However, parametric tests were initially conducted on these measures to examine the role of IQ as a covariate. The covariate did not significantly predict performance on any of the Bruce measures. Therefore, non-parametric results are reported in the text.

2 The VABS motor composite score was used for the regressions shown in Table 4 instead of the MABC-2 scores because the latter was used as an inclusion measure for group membership. Therefore, when entered into regressions the MABC-2 and Group variables were highly correlated (.90) for the TD and DCD comparisons, violating test assumptions. However, note that when the MABC-2 scores were included the same pattern of results as shown in Table 4 (a relationship between motor and socialisation for all groups) was evident.
